# Holding it together: a longitudinal study of psychological distress and associated factors in mothers with cancer

**DOI:** 10.1186/s40359-026-04332-9

**Published:** 2026-03-10

**Authors:** Andrea Hess Engström, Maria Romare Strandh, Pia Enebrink, Karin Stålberg, Lisa Ljungman, Anna Wikman

**Affiliations:** 1 Department of Women’s and Children’s Health, Uppsala University, Uppsala, Sweden; 2 Centre for Women’s Mental Health during the Reproductive Lifespan (WOMHER), Uppsala University, Uppsala, Sweden; 3 Department of Clinical Neuroscience, Karolinska Institutet, Stockholm, Sweden

**Keywords:** Parenting concerns, psychological distress, PTSD, cancer

## Abstract

**Background:**

Women with cancer tend to report higher levels of psychological distress than men with cancer. A deeper understanding of how modifiable psychological factors are related to psychological distress in mothers with cancer could inform targeted prevention strategies. The aim of this study was to investigate how the modifiable psychological factors; parenting concerns, self-efficacy, and emotion regulation strategies, are associated with symptoms of depression, anxiety, stress, and posttraumatic stress in mothers with cancer.

**Methods:**

In 2023, 222 mothers with cancer, aged 25–60 years, with varying time since diagnosis were included in a longitudinal observational study. Data were collected at inclusion and one year later. Outcome measures were symptoms of depression, anxiety, stress, and PTSD, assessed using Depression, Anxiety and Stress Scale (DASS-21) and PTSD Checklist for DSM-5 (PCL-5) questionnaires. Sociodemographic and health-related variables were used as covariates and parenting concerns, self-efficacy, and emotion regulation strategies (cognitive reappraisal and expressive suppression) were used as explanatory variables in the hierarchical linear regression models.

**Results:**

After controlling for sociodemographic and health-related variables, baseline symptom levels were the strongest predictors of symptoms of depression, anxiety, stress, and PTSD at one-year follow-up. With exception of parenting concerns in relation to depressive symptoms, modifiable psychological factors did not predict the outcomes after adjusting for the contribution of baseline symptoms.

**Conclusions:**

Baseline symptom severity emerged as a strong predictor across all outcomes, as such early assessment of psychological symptoms may be particularly important for mothers with cancer. Future research should aim to diversify the study population to better capture differences in psychological symptoms across various cancer types, cancer stage, and age groups.

## Introduction

Cancer is the second leading cause of death globally, accounting for approximately 1 in 6 deaths [1]. In 2022, an estimated 20 million new cancer cases and 9.7 million cancer-related deaths occurred worldwide [2]. One in five women is expected to develop the disease during her lifetime, and one in twelve will die from the disease [2]. Cancer imposes significant health and financial burdens [3], often with profound consequences, such as the loss of a parent during a child’s formative years [4].

Beyond its physical toll, cancer is associated with an increased risk of psychological conditions, including depression and posttraumatic stress disorder (PTSD) [5]. Sex differences in psychological distress have been reported in oncology populations, with women experiencing higher levels of anxiety and depression than men [6, 7]. Among younger women with breast cancer, stress reactions typically intensify shortly after diagnosis but tend to decrease over time [8]. Together, symptoms of depression, anxiety, stress, and posttraumatic stress may capture dimensions of psychological burden commonly experienced by women with cancer and, therefore, are central outcomes for understanding mental health in mothers coping with cancer. Several factors can exacerbate psychological distress: lower self-efficacy in managing the disease [9], poor self-rated health, higher levels of pain, and diminished physical and functional well-being [10–12]. Socioeconomic factors such as lower income and lower educational attainment are also related to greater psychological distress. Nearly two-thirds of cancer patients experience financial concerns, which further contribute to their emotional burden [13]. In Sweden, healthcare is publicly funded through the national healthcare system, and cancer treatment is heavily subsidized, meaning that patients generally do not incur direct costs for medical cancer care. Access to psychosocial services is also available within the healthcare system when clinically indicated. However, despite limited direct treatment costs, individuals on sick leave typically receive reduced income compared with regular employment, which may result in financial strain related to everyday living expenses.

Among individuals diagnosed with cancer, those who are parents of dependent children may constitute a particularly vulnerable subgroup. Compared to individuals with cancer who do not have children, parents with cancer report lower quality of life and poorer mental health [14]. In addition to the direct impact on their mental health, these parents also experience higher levels of parenting concerns [15], increased parenting stress, fear of cancer recurrence, and disruption to family life [16]. Parents with cancer often prioritize their children’s well-being while struggling to manage their own health and parenting responsibilities [17]. Lower levels of dyadic adjustment in relation to a partner and poorer family functioning are associated with greater parenting concerns and reduced parenting self-efficacy in this population [18]. While higher levels of parenting concerns are related to higher levels of symptoms of anxiety and depression; higher parenting self-efficacy is related to lower levels of these symptoms in parents with advanced cancer [19]. In women with breast cancer, concerns about their ability to fulfill roles as a parent or partner are common, while their partners frequently express worries related to the potential death of their loved one [20].

Emotion regulation strategies play a role in the psychological well-being of parents with cancer [21]. These strategies are defined as the processes individuals use to adjust their emotional reactions, experiences, and expression in order to effectively meet the demands of their environment, such as cognitive reappraisal and expressive suppression [22]. Cognitive reappraisal involves reframing emotional stimuli (changing how one thinks about a situation) that trigger emotions to decrease their impact, while expressive suppression involves inhibiting the expression of emotions [22]. A literature review has shown that, despite some inconsistencies across studies, expressive suppression is associated with increased psychological distress in patients with cancer [23]. Among mothers with cancer, low use of cognitive reappraisal has been associated with increased parenting stress [24].

Conceptually, parenting concerns, self-efficacy, and emotion regulation strategies represent interrelated factors in how mothers cope with cancer-related stressors in everyday functioning while living with cancer. Parenting concerns are understood as worries about fulfilling parental roles and protecting children’s well-being. Self-efficacy refers to mothers’ perceived ability to manage the combined demands of cancer and parenting. Emotion regulation strategies describe how emotional responses to these challenges are managed. Psychological distress in cancer populations may remain stable without intervention. However, cognitive and emotional coping processes may contribute to the persistence or attenuation of distress beyond initial symptom levels. We conceptualize parenting concerns as a chronic cancer-specific stressor, self-efficacy as a perceived coping resource, and emotion regulation strategies as mechanisms through which emotional responses are managed. Mothers experiencing high parenting concerns combined with low self-efficacy and maladaptive emotion regulation may be at risk for sustained or increasing distress over time. Conversely, higher self-efficacy and adaptive regulation strategies may buffer the impact of parenting-related stress. We have previously identified cross-sectional associations between psychological distress and greater parenting concerns, lower self-efficacy, and maladaptive emotion regulation among parents with cancer [21]. However, the cross-sectional approach limits conclusions about temporal relationships and long-term psychological outcomes. Examining these factors longitudinally allows us to determine whether they predict psychological distress beyond baseline levels.

Identifying predictors of psychological distress in this population is essential for informing the development of targeted interventions aimed at reducing psychological distress and supporting parents during and after cancer treatment. Given that women with cancer tend to report higher levels of psychological distress than men [25, 26], a deeper understanding of modifiable psychological factors in mothers coping with cancer could support more effective prevention strategies and promote better psychological outcomes in this vulnerable group. Therefore, the aim of this study was to investigate how the modifiable psychological factors; parenting concerns, self-efficacy, and emotion regulation strategies, are associated with psychological distress; that is symptoms of depression, anxiety, stress, and posttraumatic stress in mothers with cancer. By examining these associations between two time points, at inclusion and one year later, the study seeks to understand how these factors may influence symptoms of depression, anxiety, stress, and posttraumatic stress throughout the cancer trajectory.

## Materials and methods

### Study design

This study was a longitudinal observational study conducted in 2023 and 2024. The study protocol received prior approval from the Swedish Ethical Review Authority (Reference number: 2022-03088-01, date: August 29th 2022) and is reported according to STROBE guidelines.

### Participants and recruitment

The study included a convenience sample of mothers, aged 25 to 60 years, diagnosed with any type of cancer within the previous five years and having at least one child 18 years old or younger. Mothers younger than 25 years and older than 60 years were excluded to increase sample homogeneity with respect to parenting stage. Mothers over 60 years of age are less likely to have dependent children, while mothers under 25 may face distinct life-stage challenges (e.g., education or early career establishment) that could influence psychological distress in ways not directly related to parenting in the context of cancer. Recruitment occurred using various channels, including social media, patient organizations, and advertising posters distributed to the six Regional Cancer Centers in Sweden for sharing with oncology clinics in their respective regions.

### Data collection

Data collection was carried out between January 25 and May 31, 2023. The data were accessed for research purposes between March 22 and June 20, 2025. All data were collected anonymously; no identifying codes were used, and participants were not identifiable. Participants could, however, choose to provide an email address, for follow-up. Data were collected at two time-points: time of inclusion (T1), and one year later (T2). Participants completed the written informed consent and online surveys hosted on Uppsala University’s survey tool, REDCap [27]. The surveys were accessible via a project-specific website providing participants with detailed information about the study and research team. Each participant had the option to receive a gift voucher worth 200 SEK upon completing each survey.

The selection of questionnaires was informed by previous research [17, 21] and developed in collaboration with six parents who had and have had cancer. These parents helped formulate self-report questions and tested the questionnaire to ensure that it was relevant, acceptable, and could be completed within 20–30 min. A more detailed description of data collection has been published elsewhere [21].

### Outcome measures

Psychological distress was assessed by four outcomes; symptoms of (1) depression, (2) anxiety, (3) stress, and (4) posttraumatic stress. All four outcome measures were collected at both time-points (T1 and T2). Symptom levels at T2 were considered the primary outcomes capturing participants’ symptoms at one year follow-up, while corresponding T1 measures were used as covariates to control for baseline symptom severity. Symptoms of depression, anxiety and stress were assessed using the Depression Anxiety Stress Scale (DASS-21) [28]. This 21-item scale includes three subscales measuring depression, anxiety, and stress symptoms, with each item rated on a 4-point Likert scale ranging from 0 (“Did not apply to me at all”) to 3 (“Applied to me very much or most of the time”). Scores are calculated separately for each subscale, with a total score ranging from 0 to 42. The severity of symptoms is classified as normal, mild, moderate, severe, or extremely severe. To interpret the symptom severity, cut-off scores indicating at least moderate symptoms were set at ≥ 14 for depression, ≥ 10 for anxiety, and ≥ 19 for stress [29]. In this study, the Cronbach’s alpha was 0.94 for depression, 0.80 for anxiety, and 0.90 for stress.

Symptoms of posttraumatic stress were assessed using the PTSD Checklist for DSM-5 (PCL-5) [30]. This 20-item instrument measures symptoms across four subscales: intrusion, avoidance, negative alterations in cognitions and mood, and alterations in arousal and reactivity. Items are rated from 0 (“Not at all”) to 4 (“Extremely”), yielding a total score ranging from 0 to 80, with higher scores indicating greater symptom severity. A pre-established cut-off of 38 was used as a clinical threshold for probable PTSD [30]. The Cronbach’s alpha for this study was 0.96.

### Explanatory variables

Explanatory variables were modifiable psychological factors collected at T1 to support a temporal interpretation of the association between explanatory variables and the outcomes. These variables were selected based on their potential to be targeted in psychological interventions (i.e. modifiable factors).

Parenting concerns were assessed using the Parenting Concerns Questionnaire (PCQ) [31], which comprises 15 items across three subscales: practical impact of illness on the children, emotional impact of illness on the children, and concerns about co-parenting. Responses range from 1 (not at all concerned) to 5 (extremely concerned), with total scores from 1 to 5, where higher scores indicate greater parenting concerns. There is no cut-off for the PCQ; and only the total score was used, following a previous study [21], to reflect overall parenting concerns. The Cronbach’s alpha was 0.89 in this study.

General self-efficacy, defined as an individual’s belief in their ability to manage challenging situations, was measured using the General Self-Efficacy Scale (GSE) [32]. The questionnaire was used to capture participants’ overall perceived ability to manage the demands of living with cancer while caring for dependent children. This 10-item questionnaire is rated on a scale between 1 (not true at all) and 4 (exactly true), with total scores ranging from 10 to 40. Higher scores indicate greater general self-efficacy. There is no cut-off for this instrument. The Cronbach’s alpha was 0.91 in this study.

Emotion regulation was assessed using the Emotion Regulation Questionnaire (ERQ) [33]. This 10-item instrument measures two strategies: cognitive reappraisal and expressive suppression. The items are rated from 1 (strongly disagree) to 7 (strongly agree), with total scores ranging from 1 to 7. Higher scores indicate greater use of the respective emotion regulation strategy. Higher scores for cognitive reappraisal represent greater ability to change the perspective of a situation, adjusting the emotional response, while higher scores for expressive suppression represent suppressing emotions to a greater extent. There is no cut-off for the ERQ. The Cronbach’s alpha for cognitive reappraisal was 0.89 and for expressive suppression 0.74.

### Covariates

All covariates were collected at T1 to allow for examination of how baseline factors predict later outcomes. The covariates were chosen based on clinical experience and previous literature regarding factors that can potentially affect mental health among individuals with cancer and included sociodemographic and health-related variables.

Sociodemographic variables included age, education level, marital status, number of children, and age of youngest child. Health-related variables included time since diagnosis defined as years since primary diagnosis or years since last occurrence, type of cancer and cancer status. Cancer status was defined as curable or incurable. Curable cancer referred to participants who were not receiving palliative care and had received treatment with curative intent, whereas participants receiving palliative care were classified as having incurable cancer. Baseline values (T1) for the four outcome variables were also used as covariates.

### Statistical analysis

Descriptive statistics were used to summarize participant characteristics at inclusion. Results are presented as means with standard deviations (SD) or as counts (n) and percentages (%).

Univariable linear regression analyses (i.e., unadjusted analyses) were conducted to assess the association between covariates and explanatory variables with each of the outcome variables (symptoms of depression, anxiety, stress, and posttraumatic stress at T2). All outcome variables were analyzed as continuous measures. Multivariable linear regression analyses (i.e., adjusted analyses) were carried out using hierarchical models, with a separate model for each outcome variable. Covariates were included as adjustment variables, while explanatory variables were hypothesized to have a direct effect on the outcomes. All covariates and explanatory variables were retained in the models, regardless of statistical significance in the univariable analyses, based on their theoretical relevance to the outcomes. Covariates were age (years), education (secondary/post-secondary), relationship status (single/in a partnered relationship), number of children, age of the youngest child (years), time since diagnosis (years), and state of cancer (curable/incurable). Explanatory variables were parenting concerns, self-efficacy, cognitive reappraisal, and expressive suppression; all analyzed as continuous variables.

Hierarchical linear regression was conducted in three blocks. In Block 1 only the covariates were entered (sociodemographic and clinical variables). In Block 2, the baseline (T1) value of the corresponding outcome variable was added to control for initial symptom severity. Block 3, introduced the explanatory variables (parenting concerns, self-efficacy, and emotion regulation strategies) to assess their contribution beyond the effects of sociodemographic and clinical factors and baseline values of the corresponding outcome. This block order was selected to address the study’s primary aim: to determine whether baseline modifiable psychological factors predict later psychological distress.

Associations are presented as both unadjusted and adjusted β coefficients and 95% confidence intervals (CI), with a significance level set at *p* < 0.05. Multicollinearity was assessed using variance inflation factors (VIF). Overall model fit was evaluated with multiple R^2^, and autocorrelation of the residuals was assessed with the Durbin-Watson test where values close to 2 indicate that the residuals are approximately independent. All statistical analyses were performed using IBM SPSS Statistics version 28.0 (IBM Corp., Armonk, N.Y., USA).

## Results

Of a total sample of 388 mothers with cancer included in the study at T1, 222 mothers completed both T1 and T2 assessments (responders *n* = 222 [57%]; non-responders *n* = 166 [43%]). A significant difference was found between responders and non-responders regarding cancer status, with a higher percentage of non-responders having incurable cancer (39% vs. 24%, *p* < 0.001). No statistically significant differences were observed between the two groups on any other sociodemographic and health-related variables, or modifiable psychological factors. At the time of inclusion (T1), the mean age of participants was 45 years (SD = 6.12), and the mean number of children was 2.16 (SD = 0.99). The majority of mothers were in a partnered relationship (82%) and had post-secondary education (86%). Breast cancer was the most commonly reported type of cancer (77%). Participant characteristics are presented in Table 1.

**Table 1:** Participant characteristics at T1 (*n* = 222)

Sociodemographic and health-related variables	*n*	%	Mean	SD
Age (years)			45.20	6.12
Education				
Secondary education	32	14		
Post-secondary education	190	86		
Relationship status				
Single parent	40	18		
In a partnered relationship	182	82		
Number of children			2.16	0.99
Age of youngest child			10.62	5.06
Time since diagnosis at inclusion			1.38	1.41
Type of cancer				
Breast	171	77		
Gynecological	11	5		
Hematological	8	3.5		
Skin	8	3.5		
Bowel	12	5.5		
Other	12	5.5		
Cancer status				
Curable	198	89		
Incurable	24	11		
Modifiable psychological factors				
Parenting concerns (PCQ)			2.26	0.79
Self-efficacy (GSE)			28.27	5.41
Emotion regulation (ERQ)				
Cognitive reappraisal			4.54	1.15
Expressive suppression			2.94	1.22

*ERQ* Emotion Regulation Questionnaire, *GSE* General Self-Efficacy Scale, *PCQ* Parenting Concerns Questionnaire

### Variables associated with symptoms of depression, anxiety, stress, and posttraumatic stress

#### Symptoms of depression

In the unadjusted analyses, higher depression symptoms at T2 were significantly associated with lower education, higher parenting concerns, lower self-efficacy, lower cognitive reappraisal, greater expressive suppression, and higher depression symptoms at T1 (Table 2).

**Table 2:** Associations between covariates, explanatory variables and baseline depression with symptoms of depression after one year in mothers with cancer (*n* = 222)

	Depression			
	Unadjusted Unstandardized β (95% CI)	Block 1 Unstandardized β (95% CI)	Block 2 Unstandardized β (95% CI)	Block 3 Unstandardized β (95% CI)
*Covariates*				
Age (years)	-0.14 (-0.37, 0.09)	-0.04 (-0.39, 0.31)	0.10 (-0.17, 0.38)	0.12 (-0.16, 0.39)
Education				
Secondary education	*Ref*	*Ref*	*Ref*	*Ref*
Post-secondary education	**-4.31 (-8.24**,** -0.38)**	**-4.27 (-8.32**,** -0.23)**	-1.37 (-4.54, 1.80)	-1.61 (-4.79, 1.58)
Relationship				
Single parent	*Ref*	*Ref*	*Ref*	*Ref*
In a partnered relationship	1.88 (-1.74, 5.50)	1.22 (-2.53, 4.97)	2.13 (-0.78, 5.04)	2.36 (-0.56, 5.29)
Number of children	-0.11 (-1.52, 1.29)	-0.27 (-1.76, 1.22)	-0.67 (-1.83, 0.49)	-0.86 (-2.02, 0.31)
Age of the youngest child	-0.15 (-0.43, 0.13)	-0.10 (-0.51, 0.31)	-0.13 (-0.45, 0.18)	-0.13 (-0.44, 0.19)
Time since diagnosis (years)	-0.11 (-1.10,0.88)	-0.02 (-1.06,1.02)	-0.01 (-0.80,0.81)	-0.03 (-0.84, 0.78)
Cancer state				
Curable	*Ref*	*Ref*	*Ref*	*Ref*
Incurable	0.07 (-4.43, 4.56)	-0.25 (-4.79, 4.29)	-0.11 (-3.63, 3.41)	-0.76 (-4.33, 2.81)
Depression at T1	**0.69 (0.58**,** 0.80)**	--	**0.69 (0.58**,** 0.80)**	**0.62 (0.48**,** 0.75)**
*Explanatory variables*				
Parenting concerns	**4.54 (2.88**,** 6.20)**	--		**1.58 (0.03**,** 3.12)**
Self-efficacy	**-0.56 (-0.81**,** -0.32)**	--		-0.09 (-0.33, 0.15)
Cognitive reappraisal	**-1.94 (-3.12**,** -0.75)**	--		0.17 (-0.93, 1.28)
Expressive suppression	**1.84 (0.73**,** 2.96)**	--		0.20 (-0.74, 1.13)

Bold values: *p*-values < 0.05, *CI* Confidence interval

Unadjusted models: univariable model for the association between the covariate/baseline value/explanatory variable and the outcome variable

Block 1: Covariates; Block 2: Covariates + Baseline value of depression; Block 3: Covariates + Baseline value of depression + Explanatory variables

R^2^: Block 1 = 0.03; Block 2 = 0.42; Block 3 = 0.43

Durbin Watson: Block 1 = 1.93; Block 2 = 1.93, Block 3 = 1.94

VIF: Block 1 = 1.03–2.35, Block 2 = 1.03–2.37, Block 3 = 1.06–2.42

In the multivariable model including only covariates (Block 1), the model explained 3% of the variance in depressive symptoms (R² = 0.03), with higher education being statistically associated with lower depression symptoms (β = -4.27; 95% CI= -8.32, -0.23; *p* = 0.04). When depression symptoms at T1 were added to Block 2, the model explained 42% of the variance (R² = 0.42), indicating that higher symptoms at T1 were associated with higher depression symptoms at T2 (β = 0.69; 95% CI = 0.58, 0.80; *p* < 0.001). In the final model (Block 3), which included the explanatory variables, the model explained 43% of the variance (R² = 0.43). Only higher depression symptoms at T1 (β = 0.62; 95% CI = 0.48, 0.75; *p* < 0.001) and higher parenting concerns at T1 (β = 1.58; 95% CI = 0.03, 3.12; *p* = 0.046) remained statistically associated with higher depressive symptoms at T2.

No multicollinearity was detected (VIF = 1.03–2.42), and residuals were independent (Durbin-Watson = 1.93–1.98).

### Symptoms of anxiety

In unadjusted analyses, greater anxiety symptoms at T2 were associated with lower education, higher parenting concerns, lower self-efficacy, lower cognitive reappraisal, higher expressive suppression, and higher anxiety symptoms at T1 (Table 3).

**Table 3:** Associations between covariates, explanatory variables and baseline anxiety with symptoms of anxiety after one year in mothers with cancer (*n* = 222)

	Anxiety			
	Unadjusted Unstandardized β (95% CI)	Block 1 Unstandardized β (95% CI)	Block 2 Unstandardized β (95% CI)	Block 3 Unstandardized β (95% CI)
*Covariates*				
Age (years)	-0.07 (-0.23, 0.10)	-0.09 (-0.34, 0.16)	-0.18 (-0.205, 0.36)	0.18 (-0.01, 0.38)
Education				
Secondary education	*Ref*	*Ref*	*Ref*	*Ref*
Post-secondary education	**-3.89 (-6.70**,** -1.08)**	**-3.85 (-6.75**,** -0.95)**	-1.17 (-3.91, 0.49)	-1.67 (-3.88, 0.55)
Relationship				
Single parent	*Ref*	*Ref*	*Ref*	*Ref*
In a partnered relationship	0.56 (-2.05, 3.17)	0.12 (-2.57, 2.80)	0.67 (-1.35, 2.69)	0.72 (-1.31, 2.75)
Number of children	-0.09 (-1.10, 0.92)	-0.17 (-1.24, 0.89)	**-0.85 (-1.66**,** -0.04)**	**-0.89 (-1.71**,** -0.08)**
Age of the youngest child	-0.01 (-0.21, 0.19)	0.05 (-0.24, 0.35)	-0.05 (-0.27, 0.17)	-0.06 (-0.28, 0.16)
Time since diagnosis (years)	0.10 (-0.62, 0.81)	0.14 (-0.60, 0.88)	-0.16 (-0.71, 0.40)	-0.19 (-0.75, 0.38)
Cancer state				
Curable	*Ref*	*Ref*	*Ref*	*Ref*
Incurable	-0.31 (-3.54, 2.92)	-0.56 (-3.81, 2.70)	0.67 (-1.78, 3.12)	0.44 (-2.07, 2.94)
Anxiety at T1	**0.70 (0.60**,** 0.81)**	--	**0.73 (0.61**,** 0.84)**	**0.68 (0.55**,** 0.80)**
*Explanatory variables*				
Parenting concerns	**2.87 (1.66**,** 4.09)**	--		0.57 (-0.50, 1.64)
Self-efficacy	**-0.36 (-0.54**,** -0.18)**	--		-0.05 (-0.22, 0.11)
Cognitive reappraisal	**-1.16 (-2.02**,** -0.30)**	--		-0.16 (-0.91, 0.60)
Expressive suppression	**1.11 (0.30**,** 1.91)**	--		0.42 (-0.21, 1.05)

Bold values: *p*-values < 0.05, *CI* Confidence interval

Unadjusted models: univariable model for the association between the covariate/baseline value/explanatory variable and the outcome variable

Block 1: Covariates; Block 2: Covariates + Baseline value of anxiety; Block 3: Covariates + Baseline value of anxiety + Explanatory variables

R^2^: Block 1 = 0.04; Block 2 = 0.46; Block 3 = 0.47

Durbin Watson: Block 1 = 2.01; Block 2 = 1.99, Block 3 = 1.99

VIF: Block 1 = 1.03–2.35, Block 2 = 1.03–2.46; Block 3 = 1.06–2.50

The covariates-only model (Block 1), explained 4% of the variance (R² = 0.04). Higher education was associated with lower anxiety symptoms (β = -3.85; 95% CI= -6.75, -0.95; *p* = 0.01); no other covariates were statistically significant. In Block 2, which added anxiety symptoms at T1, the model explained 46% of the variance (R² = 0.46). Higher anxiety symptoms at T1 (β = 0.73; 95% CI = 0.61, 0.84; *p* < 0.001), and a lower number of children (β = -0.85; 95% CI= -1.66, -0.04; *p* = 0.04) were associated with higher anxiety symptoms. In the final model (Block 3), anxiety symptoms at T1 were the only psychological predictor (β = 0.68; 95% CI = 0.55, 0.80; *p* < 0.001). Additionally, a lower number of children was associated with higher anxiety (β = -0.89; 95% CI= -1.71, -0.08; *p* = 0.03). The model explained 47% of the variance (R² = 0.47).

Multicollinearity was not a concern (VIF = 1.03–2.50), and residual independence was confirmed (Durbin-Watson = 1.99–2.05).

### Symptoms of stress

Unadjusted analyses showed that higher parenting concerns, lower self-efficacy, lower cognitive reappraisal, greater expressive suppression, and stress symptoms at T1 were each significantly associated with higher stress symptoms. Higher education and older age were associated with lower stress (Table 4).

**Table 4:** Associations between covariates, explanatory variables and baseline stress with symptoms of stress after one year in mothers with cancer (*n* = 222)

	Stress			
	Unadjusted Unstandardized β (95% CI)	Block 1 Unstandardized β (95% CI)	Block 2 Unstandardized β (95% CI)	Block 3 Unstandardized β (95% CI)
*Covariates*				
Age (years)	**-0.22 (-0.43**,** -0.01)**	-0.15 (-0.48, 0.17)	-0.04 (-0.22, 0.30)	0.05 (-0.21, 0.31)
Education				
Secondary education	*Ref*	*Ref*	*Ref*	*Ref*
Post-secondary education	**-4.85 (-8.52**,** -1.19)**	**-4.75 (-8.50**,** -1.00)**	**-3.57 (-6.54**,** -0.61)**	**-3.59 (-6.57**,** -0.60)**
Relationship				
Single parent	*Ref*	*Ref*	*Ref*	*Ref*
In a partnered relationship	1.52 (-1.87, 4.92)	0.53 (-2.94, 4.01)	0.58 (-2.16, 3.32)	0.84 (-1.93, 3.60)
Number of children	-0.08 (-1.40, 1.24)	-0.14 (-1.52, 1.24)	-0.93 (-2.02, 0.17)	-1.02 (-2.13, 0.08)
Age of the youngest child	-0.22 (-0.48, 0.04)	-0.10 (-0.48, 0.28)	-0.07 (-0.37, 0.23)	-0.08 (-0.38, 0.22)
Time since diagnosis (years)	0.02 (-0.91, 0.95)	0.27 (-0.69, 1.23)	-0.02 (-0.78, 0.743)	-0.02 (-0.79, 0.75)
Cancer state				
Curable	*Ref*	*Ref*	*Ref*	*Ref*
Incurable	-1.33 (-5-53, 2.88)	-1.74 (-5.95, 2.47)	-0.87 (-4.20, 2.45)	-1.53 (-4.93, 1.87)
Stress at T1	**0.69 (0.58**,** 0.80)**	--	**0.68 (0.57**,** 0.80)**	**0.59 (0.43**,** 0.74)**
*Explanatory variables*				
Parenting concerns	**4.76 (3.23**,** 6.29)**	--		1.38 (-0.19, 2.95)
Self-efficacy	**-0.58 (-0.81**,** -0.35)**	--		-0.11 (-0.33, 0.12)
Cognitive reappraisal	**-1.82 (-2.94**,** -0.71)**	--		0.10 (-0.93, 1.13)
Expressive suppression	**1.52 (0.46**,** 2.57)**	--		0.37 (-0.50, 1.23)

Bold values: *p*-values < 0.05, *CI* Confidence interval

Unadjusted models: univariable model for the association between the covariate/baseline value/explanatory variable and the outcome variable

Block 1: Covariates; Block 2: Covariates + Baseline value of stress; Block 3: Covariates + Baseline value of stress + Explanatory variables

R^2^: Block 1 = 0.05; Block 2 = 0.41; Block 3 = 0.43

Durbin Watson: Block 1 = 1.93; Block 2 = 1.92; Block 3 = 1.93

VIF: Block 1 = 1.03–2.35; Block 2 = 1.03–2.39, Block 3 = 1.06–2.44

In Block 1 (covariates only), higher education remained significantly associated with lower stress (β = -4.75; 95% CI= -8.50, -1.00; *p* = 0.01). The model explained 5% of the variance (R² = 0.05). In Block 2, which included added symptoms of stress at T1, explained 41% of the variance (R² = 0.41). Symptoms of stress at T1 (β = 0.68; 95% CI = 0.57, 0.80; *p* < 0.001) were associated with higher stress symptoms. Higher education remained associated with lower stress symptoms (β = -3.57; 95% CI= -6.53, -0.61; *p* = 0.02). In Block 3 (final model), only stress symptoms at T1 and education remained statistically associated with stress symptoms at T2. Higher stress symptoms at T1 were associated with higher stress symptoms at T2 (β = 0.59; 95% CI = 0.43, 0.74; *p* < 0.001), while higher education was associated with lower stress symptoms (β = -3.59; 95% CI= − 6.57, − 0.60; *p* = 0.02). The model explained 43% the variance (R² = 0.43).

No multicollinearity was detected (VIF = 1.03–2.44), and residual independence was acceptable (Durbin-Watson = 1.92–1.93).

### Symptoms of posttraumatic stress

In unadjusted analyses, higher posttraumatic stress symptoms at T2 were associated with higher parenting concerns, lower self-efficacy, lower cognitive reappraisal, greater expressive suppression, and posttraumatic stress symptoms at T1. Higher education, older age, and having older children were associated with lower stress symptoms (Table 5).

**Table 5:** Associations between covariates, explanatory variables and baseline posttraumatic stress with symptoms of posttraumatic stress after one year in mothers with cancer (*n* = 222)

	Posttraumatic stress			
	Unadjusted Unstandardized β (95% CI)	Block 1 Unstandardized β (95% CI)	Block 2 Unstandardized β (95% CI)	Block 3 Unstandardized β (95% CI)
*Covariates*				
Age (years)	**-0.39 (-0.75**,** -0.03)**	-0.11 (-0.66, 0.44)	0.21 (-0.15, 0.57)	0.21 (-0.15, 0.58)
Education				
Secondary education	*Ref*	*Ref*	*Ref*	*Ref*
Post-secondary education	**-7.08 (-13.30**,** -0.87)**	**-7.05 (-13.39**,** -0.72)**	**-5.16 (-9.27**,** -1.04)**	**-5.11 (-9.29**,** -0.94)**
Relationship				
Single parent	*Ref*	*Ref*	*Ref*	*Ref*
In a partnered relationship	3.21 (-2.52, 8.94)	1.68 (-4.19, 7.55)	2.01 (-1.79, 5.82)	2.01 (-1.87, 5.89)
Number of children	-0.01 (-2.23, 2.22)	-0.23 (-2.57, 2.10)	-1.02 (-2.53, 0.50)	-1.02 (-2.57, 0.53)
Age of the youngest child	**-0.50 (-0.93**,** -0.07)**	-0.38 (-1.01, 0.26)	-0.25 (-0.66, 0.17)	-0.26 (-0.67, 0.16)
Time since diagnosis (years)	-0.59 (-2.15, 0.98)	-0.18 (-1.80, 1.44)	-0.74 (-1.80, 0.31)	-0.73 (-1.80, 0.34)
Cancer state				
Curable	*Ref*	*Ref*	*Ref*	*Ref*
Incurable	-2.18 (-9.29, 4.92)	-2.59 (-9.70, 4.52)	-2.51 (-7.13, 2.10)	-2.48 (-7.23, 2.27)
Posttraumatic stress at T1	**0.80 (0.71**,** 0.88)**	--	**0.79 (0.70**,** 0.88)**	**0.78 (0.65**,** 0.91)**
*Explanatory variables*				
Parenting concerns	**8.97 (6.43**,** 11.50)**	--		0.01 (-2.33, 2.35)
Self-efficacy	**-1.09 (-1.40**,** -0.62)**	--		-0.03 (-0.35, 0.29)
Cognitive reappraisal	**-2.85 (-4.72**,** -0.96)**	--		0.20 (-1.25, 1.64)
Expressive suppression	**3.78 (2.04**,** 5.52)**	--		0.45 (-0.81, 1.71)

Bold values: *p*-values < 0.05; CI=Confidence interval

Unadjusted models: univariable model for the association between the covariate/baseline value/explanatory variable and the outcome variable

Block 1: Covariates; Block 2: Covariates + Baseline value of posttraumatic stress; Block 3: Covariates + Baseline value of posttraumatic stress + Explanatory variables

R^2^: Block 1 = 0.05; Block 2 = 0.60; Block 3 = 0.60

Durbin Watson: Block 1 = 2.04; Block 2 = 2.00; Block 3 = 1.97

VIF: Block 1 = 1.03–2.35; Block 2 = 1.03–2.38; Block 3 = 1.06–2.43

In Block 1 (covariate-only), the model explained 5% of the variance (R² = 0.05). Higher education was significantly associated with lower posttraumatic stress symptoms (β = -7.05; 95% CI= -13.39, -0.72; *p* = 0.03); while age and child’s age were no longer significant. With the addition of symptoms of posttraumatic stress at T1 (Block 2), the model explained 60% of the variance (R² = 0.60). Higher symptoms of posttraumatic stress at T1 were associated with higher symptoms at T1 (β = 0.79; 95% CI = 0.70, 0.88; *p* < 0.001), while higher education (β = -5.16; 95% CI= -9.27, -1.04; *p* = 0.01) was associated with lower posttraumatic stress symptoms. In the final model (Block 3), posttraumatic stress symptoms at T1 were associated with higher posttraumatic stress symptoms (β = 0.78; 95% CI = 0.65, 0.91; *p* < 0.001). Higher education was associated with lower posttraumatic stress symptoms (β = -5.11, 95% CI= -9.29, -0.94; *p* = 0.02). Parenting concerns, self-efficacy, and expressive suppression were no longer significant. The model explained 60% of the variance (R² = 0.60).

No multicollinearity was observed (VIF = 1.03–2.43), and Durbin-Watson statistics indicated independence of residuals (1.92–2.04).

## Discussion

This study examined whether modifiable psychological factors (i.e., parenting concerns, self-efficacy, and emotion regulation strategies) were associated with symptoms of depression, anxiety, stress, and posttraumatic stress in mothers with cancer from inclusion to one year follow-up. In models that included baseline symptom severity, baseline symptoms were the strongest predictor of the corresponding symptom at follow-up across all outcomes. After accounting for baseline symptoms, most modifiable factors did not show statistically significant independent associations with T2 outcomes. Parenting concerns showed a small association with depression symptoms only. Overall, the study hypothesis was not supported as modifiable factors were mostly not associated with psychological symptoms before accounting for baseline symptom severity.

Although previous research has shown that greater parenting concerns are associated with increased psychological distress in parents with cancer [34, 35], this was largely not observed in the present study. While previous studies have demonstrated the association between parenting concerns and mental health, the independent contribution towards explaining psychological distress over time was overshadowed by prior symptom levels. Despite this finding, parenting support is a recognized need among parents with cancer, and incorporating attention to parenting concerns in clinical care could be a meaningful step toward improving well-being in this population. At present, there is limited consensus on the most effective models for addressing parenting concerns, even if some interventions have shown promise in improving mental health outcomes by targeting coping strategies and parenting self-efficacy [36].

Self-efficacy was not associated with higher symptom scores. This finding is inconsistent with prior research showing that higher parenting self-efficacy is related to lower levels of anxiety and depression in parents with cancer [19], suggesting that self-efficacy may function as a protective factor and a psychological resource. Expressive suppression was also not associated with higher symptom scores. In contrast, it has been previously reported that among patients with breast cancer, greater expressive suppression predicted higher psychological distress [37]. Conversely, parents with cancer who engage in greater emotional disclosure tend to report lower levels of depression and anxiety symptoms [20], indicating that emotion suppression may negatively affect psychological outcomes. Although self-efficacy and emotion regulation strategies did not independently predict one-year outcomes in the present study, this does not necessarily imply that they are clinically irrelevant. It is possible that these factors may be more closely related to concurrent distress or to processes targeted in therapeutic interventions, rather than functioning as independent longitudinal predictors.

Although cognitive reappraisal was associated with psychological distress in unadjusted models, these associations were no longer significant after adjusting for other variables in the models. This suggests that while cognitive reappraisal may be a helpful emotion regulation strategy, its protective effect may be context-dependent or moderated by other factors. For example, previous research found that higher cognitive reappraisal among cancer patients was associated with higher quality of life, particularly among those experiencing low and moderate social support [38]. In this study, cognitive reappraisal was moderately associated with self-efficacy (*data not shown*), suggesting that individuals who feel more capable of handling their situation may also be more likely to engage in flexible and adaptive thinking in response to their circumstances.

Results indicate that prior psychological symptoms predicted the same symptoms one year later, while higher education emerged as a protective factor, particularly for symptoms of stress and posttraumatic stress. These findings are consistent with previous research in oncology populations [25, 39–41]. The lack of associations between modifiable psychological factors and psychological distress after adjusting for baseline symptom severity suggests that prior psychological distress is a strong and stable predictor of subsequent symptoms. One possible explanation is that, once clinical symptoms are established, baseline symptoms may overshadow the independent contribution of other modifiable factors. This pattern, and our previous findings [21], indicate that modifiable factors may be particularly relevant earlier in the disease trajectory.

While education itself may not directly influence psychological symptoms, it is associated with health literacy in cancer patients [42], and often reflects broader social determinants of health [43], such as occupation, income, and access to resources, which together may reduce vulnerability. This suggests that educational level may play a role in the broader context of factors affecting psychological symptoms or serve as a proxy for multiple interrelated factors influencing psychological outcomes. Lastly, the finding that a higher number of children was associated with lower anxiety may reflect greater emotional meaning [44], which may buffer against anxiety in the context of cancer. This association may be interpreted cautiously, as it may also reflect unassessed measures in the present study, such as social support or family functioning.

### Clinical implications

The findings of this study suggest the importance of early detection of psychological distress in mothers with cancer, particularly for mothers with lower educational attainment reporting higher parenting concerns. Systematic screening at the time of diagnosis and during treatment may help to identify mothers at increased risk of persistent psychological symptoms and enable timely support.

In our previous work, we have identified cross-sectional associations between parenting concerns, self-efficacy, and emotion regulation strategies and psychological distress [21]. As such, although modifiable psychological factors did not remain significant after accounting for baseline symptoms in the present study, they may still represent relevant targets for supportive interventions, and may contribute to alleviating distress. For instance, lower levels of illness communication have been associated with lower parenting self-efficacy in parents with cancer with minor children [19], suggesting that interventions to enhance self-efficacy could empower parents to better manage both cancer-related challenges and parenting demands. Additionally, parents with higher levels of parenting concerns reported a poorer communication style [33]. Thus, interventions that promote open emotional expression and improve communication skills may be especially valuable for those experiencing depression and trauma-related symptoms. Such interventions may include structured psychoeducation, parenting-focused psychotherapy, and interventions targeting emotion regulation and communication skills, tailored to the needs of mothers with cancer. Previous research has shown that patients are often interested in receiving professional psychological support to help them talk to their children about cancer; however, many do not seek help due to a lack of awareness about available services [18]. In addition to screening and developing targeted interventions, it is essential that patients are informed about how to access psychological support in order to prevent unnecessary distress and delays in receiving appropriate care.

### Strengths and limitations

This study applied a predictive model for each outcome, incorporating clinically relevant covariates and explanatory variables. While the results do not allow causal inferences, the findings provide valuable insights into factors associated with symptoms of depression, anxiety, stress and posttraumatic stress in mothers with cancer, and can be used to inform potential clinical strategies for this population. However, the study has a number of limitations. While the use of convenience sampling enables reaching a larger population, recruitment through social media and patient organizations may have introduced selection bias. For instance, the sample was predominantly composed of highly educated mothers with breast cancer who were in a partnered relationship, which may limit the generalizability of the findings to the broader population of mothers with cancer. Mothers under 25 or over 60 years of age were excluded, leaving out mothers facing different challenges. For instance, younger cancer patients may experience higher psychological distress due to factors such as pain, changes in work routines, and poor social support [42]. Future studies should, therefore, explore younger and older populations. While retention rates one year after inclusion were reasonable, a substantial proportion of mothers with incurable cancer were non-responders, possibly due to illness burden, emotional distress, or death. Given that patients in advanced stages of cancer present with higher levels of psychosocial distress, this selective attrition may have led to an underestimation of levels of psychological distress at follow-up. Moreover, the loss of participants with potentially higher and more unstable symptom levels may have reduced variability in psychological outcomes, thereby attenuating prospective associations between psychological factors and distress. As a result, the generalizability of the associations observed in the retained sample may be limited.

Perceived social support was not assessed quantitatively in the present study due to the absence of validated measures in the study; however, it cannot be excluded that social support may have influenced the results. This is supported by a complementary qualitative study based on the same cohort [45], which highlighted the importance of broader support networks beyond the partner relationship. Self-reported information regarding psychiatric history, medication, and ongoing psychological treatment was collected; however, these variables were not included in the analyses. The study aimed to examine modifiable psychological factors related to psychological distress rather than treatment-related effects. In addition, participants were at different times since diagnosis, and engagement in medication and psychological therapy could change over time, limiting the reliability and interpretability of this variable in the present analyses. Consequently, the potential influence of psychiatric history, medication use, and psychological treatment on the outcomes cannot be ruled out and should be considered when interpreting the findings.

Finally, participants were offered a modest gift voucher (200 SEK) upon completion of each survey. While financial incentives can theoretically influence participation, the amount was intended as a symbolic token of appreciation rather than a substantive reward. It is therefore unlikely that the incentive substantially influenced participation decisions or introduced meaningful selection bias.

## Conclusion

While modifiable psychological factors were associated with symptoms of depression, anxiety, stress, and posttraumatic stress in unadjusted models, baseline symptoms severity emerged as the strongest predictors across all outcomes at follow-up. The findings underscore the importance of early identification of symptoms as these may persist over time. Future research should aim to diversify the study population to better capture differences in psychological symptoms across various cancer types, cancer stages, and age groups.

## Data Availability

The data cannot be shared publicly due to ethical restrictions and participant confidentiality under Swedish regulations. Data are available from the principal investigator, Prof. Anna Wikman (anna.wikman@uu.se), upon reasonable request for researchers who meet the criteria for access to confidential data.
